# Mitigation of Salinity Stress in Plants by Arbuscular Mycorrhizal Symbiosis: Current Understanding and New Challenges

**DOI:** 10.3389/fpls.2019.00470

**Published:** 2019-04-12

**Authors:** Heikham Evelin, Thokchom Sarda Devi, Samta Gupta, Rupam Kapoor

**Affiliations:** ^1^Department of Botany, Rajiv Gandhi University, Itanagar, India; ^2^Department of Botany, University of Delhi, New Delhi, India

**Keywords:** antioxidants, arbuscular mycorrhizal fungi, aquaporins, ionic homeostasis, photosynthetic efficiency, osmotic balance, salinity

## Abstract

Modern agriculture is facing twin challenge of ensuring global food security and executing it in a sustainable manner. However, the rapidly expanding salinity stress in cultivable areas poses a major peril to crop yield. Among various biotechnological techniques being used to reduce the negative effects of salinity, the use of arbuscular mycorrhizal fungi (AMF) is considered to be an efficient approach for bio-amelioration of salinity stress. AMF deploy an array of biochemical and physiological mechanisms that act in a concerted manner to provide more salinity tolerance to the host plant. Some of the well-known mechanisms include improved nutrient uptake and maintenance of ionic homeostasis, superior water use efficiency and osmoprotection, enhanced photosynthetic efficiency, preservation of cell ultrastructure, and reinforced antioxidant metabolism. Molecular studies in past one decade have further elucidated the processes involved in amelioration of salt stress in mycorrhizal plants. The participating AMF induce expression of genes involved in Na^+^ extrusion to the soil solution, K^+^ acquisition (by phloem loading and unloading) and release into the xylem, therefore maintaining favorable Na^+^:K^+^ ratio. Colonization by AMF differentially affects expression of plasma membrane and tonoplast aquaporins (PIPs and TIPs), which consequently improves water status of the plant. Formation of AM (arbuscular mycorrhiza) surges the capacity of plant to mend photosystem-II (PSII) and boosts quantum efficiency of PSII under salt stress conditions by mounting the transcript levels of chloroplast genes encoding antenna proteins involved in transfer of excitation energy. Furthermore, AM-induced interplay of phytohormones, including strigolactones, abscisic acid, gibberellic acid, salicylic acid, and jasmonic acid have also been associated with the salt tolerance mechanism. This review comprehensively covers major research advances on physiological, biochemical, and molecular mechanisms implicated in AM-induced salt stress tolerance in plants. The review identifies the challenges involved in the application of AM in alleviation of salt stress in plants in order to improve crop productivity.

## Introduction

Worldwide, soil salinity is becoming a significant problem as it is encountered in all climates. Amongst the various salts present in the soil, NaCl is the most prevalent one. Soils are rendered saline due to deposition of salt either by natural (primary) or anthropogenic (secondary) processes. Primary processes include weathering of parent rocks, deposition from sea water and atmospheric deposition. Secondary processes include poor drainage facilities, irrigation with brackish groundwater, continuous irrigation for long durations, improper management of water, and cultural methods in irrigated agriculture. In addition, growing annual plants with shallow root-systems in place of deep-rooted perennial flora raises the water table leading to rise of saline groundwater (Food and Agriculture Organization [FAO], 2015). It is projected that around one billion hectares (ha) traversing more than 100 countries encounter salinity problems (Food and Agriculture Organization [FAO], 2015). Soil salinity is rapidly increasing with an estimated yearly addition of 0.3–1.5 million ha of farmland thereby decreasing crop production by more than 20% (Porcel et al., 2012; Food and Agriculture Organization [FAO], 2015). It also renders another 20–46 million ha with decreased capacity for production. On the other hand, the earth is home to 7.7 billion people with addition of 83 million people every year at the rate of 1.09% (United Nations [UN], 2018). Therefore, achieving food security for the growing population amidst the declining farmland is one of the most important missions for modern agriculture.

High salt deposition in the soil results in osmotic as well as specific ion effects, which further lead to secondary oxidative stress in plants. Thus, salinity shows adverse effects on germination, growth, and reproduction of plants that subsequently diminish crop yield (Chinnusamy et al., 2005).

Plants have evolved to be a highly flexible system which can adjust its morphological, physiological, biochemical, and molecular mechanisms to survive and sustain the changing environment. To counter the problems of salinity, plants exhibit growth plasticity (morphological and developmental pattern change), accumulation of compatible osmolytes to maintain turgor as well as prevent ultrastructural damage, ion-homeostasis, regulation of water uptake and enhanced water use efficiency, enhanced photosynthesis, detoxification of ROS through antioxidant enzymes and molecules, and induction of phytohormones (Munns and Tester, 2008; Ruiz-Lozano et al., 2012; Augé et al., 2014). However, these adaptive strategies become inefficient to cope with the rapidly increasing salinity.

**FIGURE 1:**
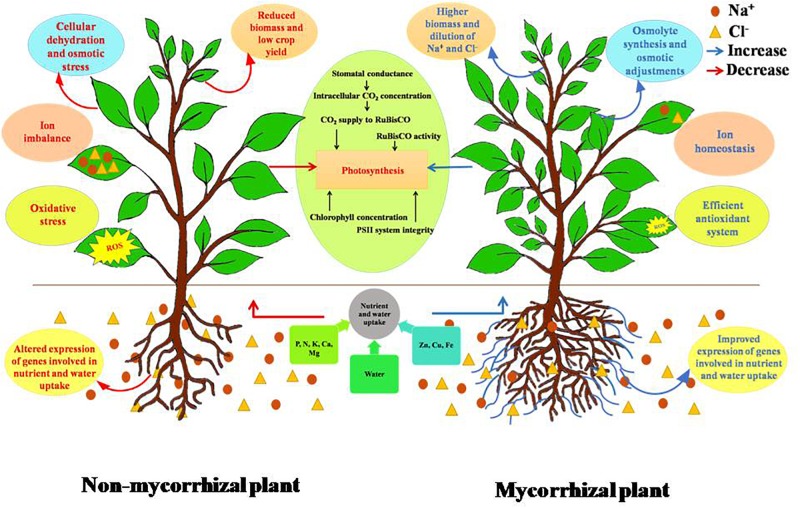
Differential response of non-mycorrhizal and mycorrhizal plants under salt stress. Accumulation of salt in soil creates competition for nutrient uptake and transport. This leads to imbalance of the ionic composition of plant, thereby affecting plant’s physiological traits. AMF increase the volume of soil explored by plant roots, upregulate several cation transporters, leading to improved nutrient uptake, and also maintains ionic homeostasis. Salinity lowers soil water potential causing cellular dehydration due to decrease in water uptake. AM negates this effect by mediating accumulation of osmolytes and also improves plant’s water status by improving root hydraulic conductivity. Salinity induces oxidative stress due to imbalance in ROS (reactive oxygen species) generation and the quenching activities of antioxidants. AMF are known to improve both enzymatic and non-enzymatic antioxidant systems of plants. Photosynthesis is also negatively affected by salinity. AM has a positive effect on photosynthesis under salt stress. Overall, AMF improve the performance of plant under salt stress.

Arbuscular mycorrhizal fungi establish a symbiotic union with roots of 80% land plants (Smith and Read, 2008). This symbiosis constitutes a distinctive system with more efficiency than roots alone for uptake and transfer of mineral nutrients from the soil (Ruiz-Lozano et al., 2012). The plant associated extramatrical hyphae of AMF can extent up to 100 m g^-1^ of soil, and enhance the plant’s ability to explore soil. These hyphae are leaner than roots, hence facilitate mining of water-filled pores, which otherwise are inaccessible to roots (Smith et al., 2010). Owing to this, AM can boost several mechanisms in plant to manage salt stress (Evelin et al., 2009; Ruiz-Lozano et al., 2012). Formation of AM has been reported to – (i) improve nutrient acquisition and maintain ionic homeostasis; (ii) improve water uptake and maintain osmotic equilibrium in plants; (iii) induce antioxidant system to prevent damage by ROS; (iv) protect photosynthetic apparatus and enhance photosynthetic efficiency; and (v) modulate phytohormone profile to minimize salt effects on growth and development (Figure 1) (Evelin et al., 2009, 2012; Ruiz-Lozano et al., 2012; Augé et al., 2014; Khalloufi et al., 2017). These effects act in coordination to improve plant’s resilience to salinity stress. These ameliorative effects can be evaluated in terms of improved plant growth exhibited by mycorrhizal (M) in comparison to NM plants. In the past decade, significant progress has been made to understand these mechanisms. This review comprehensively covers all biochemical and physiological changes that occur in plants that are inoculated with AM fungi and exposed to salt stress. It essentially takes stock of the advances made in the last decade toward better understanding of the mechanisms that contribute to salt stress alleviation in M plants. These include molecular bases for higher K^+^:Na^+^ ratio and higher concentration of N in M plants via regulation of transporters; maintenance of efficient water status via differential regulation of aquaporin genes (PIPs and TIPs); and elucidation of better photosynthetic efficiency via upregulation of RuBisCo gene as well as protection of PSII by upregulation of genes encoding D1 and D2 proteins of PSII. It updates the role of AM in influencing interplay of phytohormones in plants subjected to salt stress. Finally, it identifies the gaps in the understanding of the mechanisms, and presents the research challenges to be met in the future studies.

## Mechanisms of Salt Tolerance in M Plants

The ability of plants to tolerate salinity stress is usually evaluated in terms of biomass produced (da Silva et al., 2008; Ruiz-Lozano et al., 2012). Several studies have highlighted that AMF imparts salinity tolerance in host plants by virtue of higher biomass as compared to NM plants. AMF colonization enhanced biomass in *Trigonella foenum-graecum* (Evelin et al., 2012), *Oryza sativa* (Porcel et al., 2015), *Medicago sativa* (Campanelli et al., 2013), *Gossypium hirsutum* (Liu et al., 2016), *Elaeagnus angustifolia* (Chang et al., 2018), and *Chrysanthemum morifolium* (Wang et al., 2018). Higher biomass subsequently leads to dilution of Na^+^ and Cl^-^, and manifests as better crop yield (Talaat and Shawky, 2011).

### Alteration in Root Architecture

Root system, being the plant structure responsible for uptake of water and nutrients, is crucial for enhancing plant resistance to salt stress. It is also the organ that regulates salt acquisition and translocation (Jung and McCouch, 2013). In the presence of salt in the rhizosphere, plants experience a decline in primary root growth due to salt-induced inhibition of cell division and elongation of root epidermal cells while lateral root development gets initiated (Rahnama et al., 2011; Jung and McCouch, 2013). In fact, it is well established that AMF colonization can improve plant’s adaptive ability by modifying the structure of root according to the requirements of time and space (Kapoor et al., 2008). Wu et al. (2010) observed that the length, surface area, and projected area of the root were more in M than the NM *Citrus* plants. Kumar et al. (2010) reported greater root length and biomass in M than NM *Jatropha curcas* plants. Similar observations have been reported in *Trigonella foenum-graecum* (Evelin et al., 2012), *Medicago sativa* (Campanelli et al., 2013), *Ephedra aphylla* (Alqarawi et al., 2014), and *Cucurbita pepo* var. *pepo* (Harris-Valle et al., 2018). Better root system enables the plant to track non-saline areas for water and minerals until exploitation of salt areas become indispensable (Wu et al., 2010; Campanelli et al., 2013; Alqarawi et al., 2014).

### Nutrient Acquisition and Ionic Homeostasis

Excess salt (Na^+^ and Cl^-^) in the soil affects availability of nutrients by imposing competition during uptake, translocation or apportioning within the plant (Rabie, 2005). Therefore, high Na^+^ and Cl^-^ concentrations in the soil solution may suppress nutrient associated activities and result in undesired ratios of Na^+^:Ca^2+^, Na^+^:K^+^, and Ca^2+^:Mg^2+^ (Abdel-Fattah and Asrar, 2012). Such a phenomenon can lead to imbalance in ionic composition of the plant, thereby affecting plant’s physiological traits (Hasegawa et al., 2000; Munns et al., 2006). However, AMF colonization has been shown to improve nutrient uptake and maintain ionic homeostasis in host plants grown in saline soils (Table 1). In fact, it has been assessed that the extramatrical hyphae of AMF can supply up to 80, 25, 10, 25, and 60% of plant’s P (phosphorus), N (nitrogen), K (potassium), Zn (zinc), and Cu (copper), respectively (Marschner and Dell, 1994). AMF colonization also influences the concentration and profile of organic acids and polyamines in plants (Sheng et al., 2011; Evelin et al., 2013; Talaat and Shawky, 2013). Organic acids play important role in lowering soil electrical conductivity and increasing the availability of N, P, and K in soil (Sheng et al., 2011). Polyamines help in retaining ion homeostasis in plant cells by enhancing the uptake of nutrients and water (Pang et al., 2007).

**Table 1:** Some of the studies on effect of salinity and AMF on nutrient concentration and ionic ratios in plants.

S. No.	Salt level (mM NaCl)	Plant (Family)	Fungus^∗^	Parameters	Effects		References
						
					Salinity	AMF on salt stressed plants	
1.	0, 100	*Citrus tangerine* (Rutaceae)	*Glomus mosseae* and *Paraglomus occultum*	Na, K, Ca, Mg content and K^+^:Na^+^, Ca^2+^:Na^+^, Mg^2+^:Na^+^ ratios	Increased- Na, Ca, and Mg content, decreased- K content and ionic ratios	Increased- K, Mg content and ionic ratios, Ca^2+^ content increased by *P. occultum* Decreased- Na and Ca content, Ca^2+^:Na^+^ ratio by *G. mosseae* only	Wu et al., 2010
2.	0, 50, 100, 200	*Trigonella foenum- graecum* (Fabaceae)	*Glomus intraradices*	Shoot and root nutrient status and K^+^:Na^+^, Ca^2+^:Na^+^, Ca^2+^:Mg^2+^ ratios	Increased- shoot Na, Mn and root Na, Mg content Decreased- shoot and root N, P, K, Ca, Cu, Fe, Zn content, and ionic ratios	Increased- root and shoot N, P, K, Ca, Mg, Cu, Fe, Mn, Zn content and K^+^:Na^+^ ratio Decreased- root and shoot Na content	Evelin et al., 2012
3.	0, 50, 100, 150, 200	*Medicago sativa* (Fabaceae)	*Glomus viscosum*	Shoot and root Na, K, Ca, Mg, Cl content.	Increased- Na and Cl content Decreased- K, Ca, and Mg content	Increased- K and Mg content Decreased- Na, Ca, and Cl content	Campanelli et al., 2013
4.	0, 100	*Kosteletzkya virginica* (Malvaceae)	*G. mosseae*	Root and shoot N, P, K content and ionic ratios of different root tissues	Increased- K^+^:Na^+^ and Ca^2+^:Mg^2+^ratios Decreased- root and shoot N, P, K content, Na^+^:Ca^2+^ ratio	Increased- root and shoot N, P, K content, Na^+^:Ca^2+^ ratio Decreased- K^+^:Na^+^ and Ca^2+^:Mg^2+^ ratios	Zhang et al., 2014
5.	0, 75, 150	*Oryza sativa* (Poaceae)	*Claroideoglomus etunicatum*	Root and shoot P, Na and K content and expression of genes with a role in the uptake, transport or compartmentation of Na^+^ and/or K^+^	Increased- root and shoot Na content, Na root-to-shoot distribution Decreased- root and shoot K^+^:Na^+^ ratio, expression of *OsNHX3, OsSOS1, OsHKT2;1*, and *OsHKT1;5* genes	Increased- root Na content, root and shoot P, upregulation of *OsNHX3, OsSOS1, OsHKT2;1*, and *OsHKT1;5* genes Decreased- shoot Na content, root and shoot K^+^:Na^+^ ratio, root-to-shoot distribution of Na	Porcel et al., 2016
6.	0, 60, 80, 100	*Cicer arietinum* (Fabaceae)	*Funneliformis mosseae*	Nutrient status and K^+^:Na^+^ ratio	Increased- Na content Decreased- N, P, K, Mg content and leaf and root K^+^:Na^+^ ratio	Increased- N, P, K, Mg content, leaf and root K^+^:Na^+^ ratio Decreased- Na content	Garg and Bhandari, 2016b
7.	0, 200	*Cucumis sativus* (Cucurbitaceae)	*C. etunicatum, Rhizoglomus intraradices, and G. mosseae*	Ionic status and electrolytic leakage	Increased- Na content and electrolytic leakage Decreased- K, Ca, Mg, Fe, Zn, Mn, and Cu content	Increased- K, Ca, Mg, Fe, Zn, Mn, and Cu content Decreased- Na content and electrolytic leakage	Hashem et al., 2018


*^∗^Nomenclature of the fungus is as per the name used in the cited papers. *OsNHX3, Oryza sativa* sodium/hydrogen exchanger; *SOS1*, salt overly sensitive; *HKT*, high affinity potassium transporter.*

#### Phosphorus

Salinization renders P unavailable to plants due to its precipitation with other cations, such as Ca^2+^, Mg^2+^, and Zn^2+^ depending upon the pH of the soil environment (Azcon-G de Aguilar et al., 1979), thereby creating salt-induced P deficiency in plants. This results in stunted growth of the plant and the older leaves die prematurely (Taiz and Zeiger, 2006). However, AMF can significantly improve P acquisition for better growth and development of host plant (Table 1). Enhanced P acquisition in M plants is attributed to – (i) increased availability of P in the soil due to secretion of acid and alkaline phosphatases by hyphae that liberates P from its bound form; (ii) maintenance of intrinsic phosphate concentration (Pi) by forming polyphosphates inside the hyphae; (iii) ability of AMF to take up P at lower threshold owing to the expression of high affinity phosphate transporter genes (*GvPT, GiPT*, and *GmosPT*); and (iv) sustained movement of P into the roots as AMF are capable of accumulating vast amounts of absorbed P than roots (Bolan, 1991; Marschner and Dell, 1994; Selvaraj and Chellappan, 2006; Abdel-Fattah and Asrar, 2012). Thus in M plants, effective P uptake aids in – (i) preserving the integrity of cell membrane; (ii) reducing leakage of ions; (iii) compartmentalization of toxic ions in vacuoles and; (iv) selective uptake of ions (Rinaldelli and Mancuso, 1996; Evelin et al., 2012), consequently reducing the adverse effects of salinity.

#### K^+^:Na^+^ Ratio

Sodium and potassium ions, due to their similar physico-chemical nature, compete at the transport sites for entry into the symplast. Therefore, in saline soils where concentration of Na^+^ in rhizosphere is very high, K^+^ uptake faces a stiff competition from Na^+^, eventually decreasing K^+^:Na^+^ ratio in the cytosol. Low K^+^:Na^+^ ratio in the cell subsequently disrupts protein synthesis, enzyme activity, photosynthesis, turgor maintenance, and stomatal movement (Maathuis and Amtmann, 1999). The integrity and selectivity of root membrane are also altered by Na^+^ (Grattan and Grieve, 1999).

High Na^+^:K^+^ ratio in plants indicates a higher level of stress. Therefore, plants must consistently maintain low Na^+^:K^+^ to be able to resist the deleterious effects of salinity (Evelin et al., 2012). One of the significant advantages of favorable K^+^:Na^+^ ratio is the protection of photosynthetic tissues by inhibition of Na^+^ entry into them, a pivotal trait involved in overcoming salinity stress in glycophytes (Colmer et al., 2006; Munns and Tester, 2008; Cuin et al., 2011). In fact, photosynthetic organs constitute a major site for maintaining desirable K^+^:Na^+^ ratio. This, in turn, determines the photosynthetic ability, and hence development and productivity of the plant under saline conditions (Wu et al., 2013).

Mycorrhizal plants have unfailingly shown higher K^+^:Na^+^ ratio than their NM counterparts under salt stress conditions atleast in reported studies (Table 1). M plants can control Na^+^ translocation to aboveground parts as well as regulate internal concentrations of Na^+^. This is attributed to M plant’s ability to sequester Na^+^ into the vacuoles or exclude it from the cytosol. The toxic effects of Na^+^ in apoplast are lesser with respect to cytoplasm. Furthermore, AM facilitates host plant to retract Na^+^ from xylem, and divert it away from photosynthetic tissues to roots (Evelin et al., 2012; Maathuis, 2013). In this regard, it is interesting to note that, M plants have an added advantage over their NM counterparts as salinity induces accumulation of glomalin in participating AMF (Hammer and Rillig, 2011). Glomalin, a glycoprotein has been described as a heat shock protein 60 (HSP60) homolog (Gadkar and Rillig, 2006) and is hypothesized to participate in lowering cytosolic damages due to Na^+^-mediated protein misfolding (Maathuis and Amtmann, 1999). The strong correlation between salinity and production of glomalin only strengthens our understanding of AM protection of plants under salt stress (Hammer and Rillig, 2011).

**FIGURE 2:**
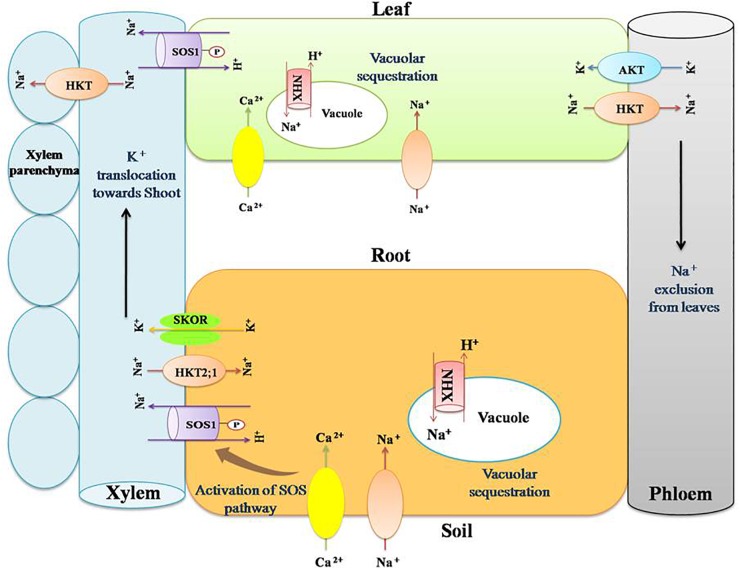
Role of transporter proteins in maintaining favorable K^+^:Na^+^ ratio in salt stressed plants. Salinity renders more concentration of Na^+^ and Cl^-^ in the soil. This results in imbalance in the ion uptake by plants. Na^+^ and K^+^ have similar physico-chemical nature and hence compete at their transport sites for entry into root symplast. Salt stress results in higher Na^+^ uptake and thus its cellular composition increases, leading to disruption of enzyme activity, protein synthesis, turgor maintenance, and so on. Plants counteract such negative effects by maintaining lower cellular Na^+^ content by activating certain cation transporters such as NHXs (sodium/hydrogen exchanger), SOS (salt overly sensitive), SKOR (outward rectifying K^+^ channel), HKT (high affinity potassium transporters), and AKT (inward rectifying K^+^ channel). NHXs are vacuolar Na^+^/H^+^antiporters. They help in maintaining lower cellular Na^+^ content by sequestering Na^+^ inside the vacuole. SOS1 is a plasma membrane Na^+^/H^+^ antiporter that extrude Na^+^ from the cytosol. It helps in reallocation of Na^+^ in roots and shoots. HKTs are Na^+^/K^+^ transporters that act in removing Na^+^ from the xylem stream and translocating it into the xylem parenchyma. SKOR mediates translocation of K^+^ toward shoot through xylem. AKT is a K^+^ channel present in phloem and mediate influx of K^+^ to shoots.

Recent studies have explained the molecular bases of high K^+^:Na^+^ ratio in M plants (Asins et al., 2013; Porcel et al., 2016; Chen et al., 2017). Mycorrhizal *Oryza sativa* plants were able to compartmentalize Na^+^ into the vacuole via up regulation of *OsNHX3* (sodium/hydrogen exchanger), and mediate efflux of Na^+^ from cytosol to apoplastic spaces via higher expression of *OsSOS1* (salt overly sensitive) and *OsHKT2;1* (high affinity potassium transporter) (Porcel et al., 2016). NHXs are vacuolar Na^+^/H^+^ antiporters present in roots and leaves that help in sequestering Na^+^ in the vacuole (Munns, 2005). SOS1 are plasma membrane Na^+^/H^+^ antiporters responsible for secretion of Na^+^ from the cytosol beyond plasma membrane, their reallocation in roots and shoots, and restraining them from getting into the photosynthetic organs (Munns, 2005; Olías et al., 2009). HKT are Na^+^/K^+^ transporters responsible for diverting Na^+^ from photosynthetic tissues to roots, and confiscating Na^+^ from the xylem (Figure 2) (Davenport et al., 2007). It is proposed that the functions of SOS1 and HKT are synchronized to bring Na^+^ homeostasis and apportioning among the plant organs (Pardo et al., 2006; Olías et al., 2009). Moreover, the level of stress is crucial in regulating the expression of a HKT gene (Porcel et al., 2016). For instance, *OsHKT1;5* gene expression increased seven-fold in M rice plants at 75 mM NaCl; however, its expression remained more or less same in NM plants at higher salinity level (Porcel et al., 2016).

In *Robinia pseudoacacia* roots, under salinity stress, AM symbiosis enabled exclusion of Na^+^ from root cells, unloading of Na^+^ from xylem, and translocation of K^+^ to the shoots. These effects were associated with the up regulation of root *RpSOS1, RpSKOR*, and *RpHKT1* (Chen et al., 2017). SKOR (outwardly rectifying K+ channel) is involved in the translocation of K^+^ toward shoot through xylem (Figure 2) (Gaymard et al., 1998).

#### Nitrogen

Plants absorb N as nitrate (NO_3_^2-^) and ammonium (NH_4_^+^) ions (Frechilla et al., 2001). However, salinity conditions interfere with their uptake by immobilizing them (Hodge and Fitter, 2010; Miransari, 2010). While NO_3_^2-^ uptake is challenged by Cl^-^, NH_4_^+^ absorption faces competition from Na^+^ at the membrane. Salt-induced disruption of membrane proteins that change plasma membrane integrity also affects the uptake of NO_3_^2-^ and NH_4_^+^ (Köhler and Raschke, 2000). This competition results in a low flux of NO_3_^2-^ from soil to roots leading to reduced activity of NR, as it is a substrate-inducible enzyme (Hoff et al., 1992). Several studies have reported that AMF colonization helps in increasing N uptake under stress conditions (Table 1). In fact, AMF hyphae have been reported to supply up to 25% of plant’s N (Marschner and Dell, 1994). Improved nitrate uptake in M plants is attributed to AMF-facilitated maintenance of membrane stability and increased NR activity (Talaat and Shawky, 2014). Higher NR activity in M plants can be explained by – (i) the sustained phosphate supply to the enzyme (Ruiz-Lozano and Azcón, 1996); (ii) a higher flux of nitrate (substrate), mediated by extramatrical mycelium of AMF; and (iii) regulation of NR activity (Kaldorf et al., 1998). Recently, Felicia et al. (2017) demonstrated that higher nitrogen uptake in M plants under salt stress is due to higher expression of nitrate (*NRT1.1, NAR2.2*) and ammonium transporters (*AMT1.1 and AMT1.2*) in M durum *Triticum aestivum* plants (colonized with a mixture of *Rhizophagus irregularis* and *Funneliformis mosseae*). NRT1.1 is a dual affinity transporter protein that is responsible for both low and high affinity nitrate uptake in roots (Crawford and Glass, 1998; Wang et al., 1998; Liu et al., 1999) while NAR2.2 is a protein that takes part in HATs (high affinity transporter systems) by actively interacting with the genes involved in effective NO_3_^2-^ transport (Filleur et al., 2001). However, in a prior study, it was observed that AMF symbiosis significantly up regulated the expression of *NRT1.1, NRT2, NAR2.2, AMT1.2*, and *AMT2.1* in durum *Triticum aestivum* only when grown under N-limiting conditions (Saia et al., 2015).

#### Ca^2+^:Na^+^ and Ca^2+^:Mg^2+^ Ratio

One of the parameters to measure salt stress in plants is to determine Ca^2+^:Na^+^ ratio. Under salt stress, an elevated Na^+^ concentration in the rhizosphere hampers the absorption of Ca^2+^ by replacing them in cell wall and plasma membrane. This results in translocation of less Ca^2+^, thereby reducing Ca^2+^:Na^+^ ratio in salt stressed plants (Grattan and Grieve, 1999). Low Ca^2+^:Na^+^ ratio decreases hydraulic conductivity and cell turgor, and disturbs Ca^2+^ signaling (Läuchli and Lüttge, 2002). Ca^2+^ uptake is also challenged by the presence of Mg^2+^. Magnesium is a macronutrient and is the central ion of the chlorophyll molecule. It is responsible for harvesting light for photosynthesis. It is also required for proper functioning of many enzymes, such as RNA polymerases, phosphatases, carboxylases, ATPases, protein kinases, and glutathione synthase (Shaul, 2002). Several studies have reported that Mg^2+^ concentration in plants decreases under salt stress conditions (Table 1). However, M plants possess higher concentration of Mg^2+^ than NM plants. It is interesting to note that M plants, despite accumulating more Mg^2+^ than NM plants, are also shown to have desirable Ca^2+^:Na^+^ ratio by improving Ca^2+^ uptake under salt stress conditions (Evelin et al., 2012). This AM effect is yet to be understood and requires further studies to unravel the mechanisms involved.

#### Micronutrients

The acquisition of micronutrients (Zn, Cu, Fe) by plants is exceedingly affected by salinity (Grattan and Grieve, 1992). Salinity decreases the solubility and mobility of micronutrients, such as Cu and Fe (Grattan and Grieve, 1999), thereby creating a depletion zone around the root. The depletion zone around the root results in decrease in uptake of the micronutrients by plants. However, M plants showed higher concentration of these micronutrients than NM plants (Table 1). This may be credited to – (i) widespread root-hyphal system that shortens the path of nutrients’ entry into plant (Subramanian et al., 2009); (ii) fungal mycelium serving as a substrate for nutrients to bind; (iii) AMF-induced changes in the pH of the rhizosphere, which modulates nutrient solubility and thus their availability (Li and Christie, 2001); (iv) increase in sink size of Cu and Zn due to higher shoot P, which subsequently instigate nutrient uptake and translocation to shoots (Liu et al., 2000); (v) up regulation of the expression of transporter gene of these nutrients, for example, a plasma membrane Zn transporter gene, *MtZIP2* is up regulated upon colonization by AMF (Burleigh et al., 2003).

The role of AMF in maintaining ionic balance is being elucidated at molecular level. However, much remains to be investigated on AMF-influenced higher Ca^2+^:Na^+^, Ca^2+^:Mg^2+^ ratios as well as uptake of micronutrients.

### Osmoregulation

**FIGURE 3:**
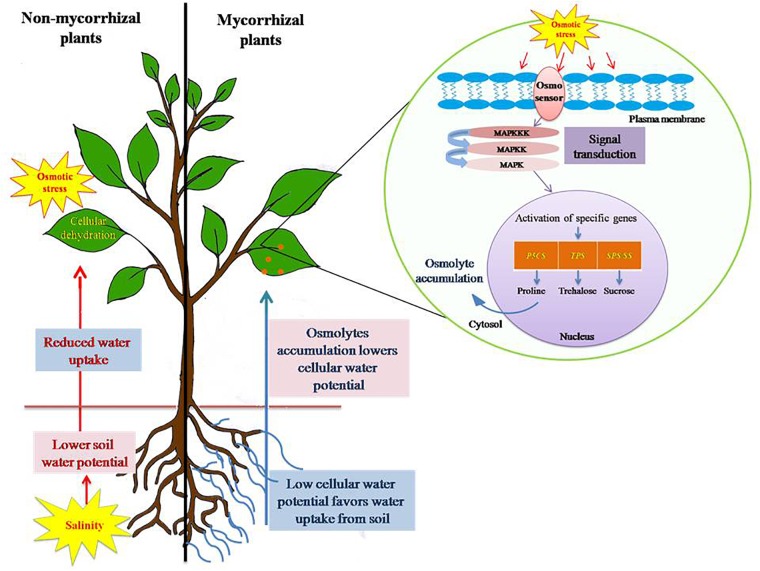
Salinity stress induced osmotic stress tolerance mechanism in plants. Salinity leads to build up of Na^+^ and Cl^-^ in soil, consequently lowering the soil water potential as compared to water potential of plant cells. This leads to reduced water uptake by plants and eventually causes cellular dehydration. Plants, in order to avoid such consequences, accumulate osmolytes, such as proline, trehalose, polyamines, and sucrose in higher concentration. Osmolytes accumulation results in lowering of cellular water potential and thereby maintains a favorable gradient for water uptake from soil to root. Thus, it prevents cellular dehydration and subside osmotic stress caused by salinity. AM symbiosis alleviates osmotic stress by influencing the expression of specific genes (*P5CS*, pyrroline-5-carboxylate synthase; *TPS*, trehalose-6-phosphate synthase; *SPS*, sucrose phosphate synthase; *SS*, sucrose synthase) involved in the biosynthesis of osmolytes.

Salt stress causes cellular dehydration by lowering turgor pressure in plant cells. In order to negate this effect, plants employ osmoregulation as a mechanism to tolerate salt stress (Munns, 1993; Zou et al., 2013). The buildup of Na^+^ and Cl^-^ in soil decreases the water potential of the soil. In such a condition, plants must retort to lower water potential in order to maintain a favorable gradient for water flow from soil into roots, and prevent dehydration of cells. To accomplish this, plants start to accumulate osmolytes, such as proline, betaine, polyamines, sugars, organic acids, amino acids, and trehalose. Osmolytes are small organic solutes that are water soluble and non-toxic at high concentrations and are also known as compatible solutes (Chen and Murata, 2011). Under salt stress, M plants have been shown to possess higher osmotic potential than their NM counterparts (Navarro et al., 2014) due to accumulation of more osmolytes (Figure 3) (Evelin et al., 2013; Garg and Baher, 2013; Alqarawi et al., 2014). The fundamental role of these osmolytes is osmotic adjustment (Hasegawa et al., 2000) In addition, they are also involved in ROS quenching, maintenance of membrane integrity, and enzyme as well as protein stabilization, hence are also known as osmoprotectants (Ashraf and Foolad, 2007). The osmolytes and their role in improving salt tolerance in M plants are discussed subsequently.

#### Proline

Salt stress tolerance mechanisms often include accumulation of proline. Proline can scavenge ROS and stabilize DNA, proteins and membranes, and reduce NaCl-induced enzyme denaturation (Kaur and Asthir, 2015). In addition, it also delivers carbon, nitrogen, and energy. This, in turn, supports plant during stress and aids in its recovery from stress. The reports on impact of AMF on proline concentration in salt stressed plants have been inconsistent. Many studies reported higher proline content in M plants compared to NM plants, while others have reported lower proline content in M plants (Table 2). Higher proline content in M plants has been attributed to – (i) increase in the expression of gene encoding *P5CS* involved in proline biosynthesis; (ii) higher activity of the enzyme P5CS; (iii) higher activity of the enzyme, glutamate dehydrogenase, that is responsible for synthesizing glutamate, the precursor of proline; and (iv) inactivation of proline dehydrogenase, an enzyme that catalyze the degradation of proline (Abo-Doma et al., 2016). Besides its role as an osmoprotectant, proline is also considered as a stress marker. Therefore, M plants may accumulate less proline as they experience reduced stress (Rabie and Almadini, 2005; Sannazzaro et al., 2007).

**Table 2:** Some of the studies on effect of salt stress and AMF on osmotic regulation in plants.

S. No.	Salt level (mM NaCl)	Plant (Family)	Fungus^∗^	Parameters	Effects of		References
						
					Salinity	AMF on salt stressed plants	
1.	0, 100, 200, 300	*Pennisetum glaucum* (Poaceae)	*Glomus fasciculatum*	Shoot and root proline content	Increased	Increased- root proline level Decreased- shoot proline level after 90 days	Borde et al., 2011
2.	0, 50, 100, 200	*Trigonella foenum-graecum* (Fabaceae)	*Glomus intraradices*	Proline, GB, polyamines, and TSS content	Increased- proline, GB, TSS, putrescine, spermidine, and spermine content	Increased- GB, TSS, spermidine, and spermine content Decreased- proline and putrescine content	Evelin et al., 2012
3.	0, 50, 100, 200	*Capsicum annum* (Solanaceae)	*Glomus intraradices*	Leaf and root proline, TSS, and reducing sugar content	Increased- leaf and root proline content Decreased- TSS and reducing sugar content	Further increased- leaf and root proline, TSS; root reducing sugar content Decreased- leaf reducing sugar	Beltrano et al., 2013
4.	0, 66, 100	*Zea mays* (Poaceae)	*Rhizophagus intraradices, Septoglomus constrictum*, and *Claroideoglomus etunicatum*	Root and shoot proline content	Increased	Increased- shoot proline by *C. etunicatum* at 100 mM only, decreased- root and shoot proline at 66 mM and 100 mM by *R. intraradices* and *S. claroideum*; *C. etunicatum* decreased root proline content	Estrada et al., 2013
5.	0, 250	*Panicum turgidum* (Poaceae)	*Funneliformis mosseae, R. intraradice*s, and *C. etunicatum*	Proline and GB content	Increased	Decreased	Hashem et al., 2015
6.	0, 60, 100	*Cajanus cajan* (Fabaceae)	*F. mosseae* and *Rhizophagus irregularis*	Trehalose metabolism	Increased- trehalose content, trehalose-6-phosphate synthase and trehalose-6-phosphate phosphatase activity Decreased-trehalase activity	Further increased- trehalose content, trehalose-6-phosphate synthase and trehalose-6-phosphate phosphatase activity Further decreased- trehalase activity	Garg and Pandey, 2016
7.	0, 100	*Cenostigma midale* (Fabaceae)	*Acaulospora longula* and *C. etunicatum*	Proline, TSS, sucrose, and fructose content	Increased- proline and TSS content	Further increased- proline and fructose content Decreased- sucrose and TSS content	Frosi et al., 2016


*^∗^Nomenclature of the fungus is as per the name used in the cited papers. TSS, total soluble sugar; GB, glycinebetaine.*

#### Trehalose (Tre)

Trehalose (α-D-glucopyranosyl-1,1-α-D-glucopyranoside) is a non-reducing storage disaccharide that regulates carbohydrate metabolism (Lunn et al., 2014). It acts as a stress protection metabolite by maintaining K^+^:Na^+^ ratio, scavenging ROS, and increasing the concentration of soluble sugars in plants (Garg et al., 2002; Redillas et al., 2012; Chang et al., 2014). Salinity enhances the buildup of trehalose and AM symbiosis can further boost the accumulation of this osmolyte. Garg and Pandey (2016) reported that M plants under salt stress accumulate more trehalose than their NM counterparts. Higher trehalose concentration in M plants can be attributed to AMF-facilitated increased activities of TPS and TPP and lower activity of TRE (Garg and Pandey, 2016). TPS and TPP are enzymes responsible for the biosynthesis of trehalose while TRE is a trehalose degrading enzyme.

#### Organic Acids

Organic acids are important osmolytes in plant vacuoles, and the regulation of their metabolism plays an important role in providing tolerance to salt stress (Guo et al., 2010). AMF colonization can also influence the concentration and profile of organic acids in plants. Under salt stress, M *Zea mays* plants accumulated more of acetic, citric, fumaric, malic, and oxalic acids, whereas concentration of formic acid and succinic acid decreased, while there was no difference in lactic acid concentration as compared to NM plants (Sheng et al., 2011). However, the mechanisms underlying changes in organic acids in M plants are not known and calls for investigation. It is speculated that AMF impart protection to the enzymes involved in organic acid biosynthesis (Sheng et al., 2011).

#### Polyamines

Polyamines are aliphatic, low molecular weight polycations that have been indicated to participate in cellular osmoregulation in plants under salt stress (Duan et al., 2008; Kuznetsov and Shevyakova, 2010). Under salt stress, polyamines accumulate in plants as compatible solutes (Minocha et al., 2014; Singh et al., 2015; Yang and Guo, 2017; Zapata et al., 2017). Major polyamines that accumulate under salt stress are putrescine (Put, diamine), spermidine (Spd, triamine), and spermine (Spm, tetraamine) (Pang et al., 2007; Kusano et al., 2008; Minocha et al., 2014). However, it remains unclear as to which polyamine is responsible more in imparting salt tolerance. AMF has also been found to modulate polyamine pool to help plants tolerate salt stress (Sannazzaro et al., 2007; Evelin et al., 2013; Talaat and Shawky, 2013). Mycorrhizal plants had higher Spd+Spm:Put ratio than NM *Trigonella foenum-graecum* plants (Evelin et al., 2013). The effect of AM on individual polyamine varies with genotype and intensity of stress (Sannazzaro et al., 2007). However, the mechanism underlying AMF-facilitated modulation of polyamines for salt tolerance in plants is yet to be deciphered, and further investigations should focus polyamines metabolism.

#### Sugars

Accumulation of TSSs such as, glucose, sucrose, dextrins, and maltose in salt stressed plants is another way of osmotic adjustment. They play a vital role in osmoprotection and as carbon storage (Parvaiz and Satyawati, 2008). Plants direct the synthesis of TSS from starch and sucrose by upregulating the activities of sucrose anabolizing enzymes. Starch is converted into dextrins and maltose by α- and β-amylases, respectively (Schrader and Sauter, 2002; Ballicora et al., 2004). SPS and SS catalyze the synthesis of sucrose while AI catalyzes the breakdown of sucrose to glucose (Van den Ende et al., 2002; Peng et al., 2016). During salt stress, sucrose undergoes decomposition in order to meet the requirement of glucose (Geigenberger and Stitt, 1993). Salt stress induced TSS accumulation is further enhanced by AM symbiosis (Talaat and Shawky, 2011; Liu et al., 2016; Garg and Bharti, 2018; Zhu et al., 2018). The higher accumulation of TSS in M plants has been credited to – (i) higher photosynthetic efficiency; (ii) higher activities of α- and β-amylases, SS, and AI; (iii) higher organic acid content; and (iv) carbon requirement of AMF (Garg and Baher, 2013; Yu et al., 2015; Zhu et al., 2016, 2018). Garg and Bharti (2018) reported that salt tolerant M *Cicer arietinum* cultivar (PBG 5) accumulated more sugars than its salt sensitive M counterparts (BG256) indicating that accumulation of TSS can enhance tolerance to salt stress. This is accompanied by higher activities of α- and β-amylases in M plants, indicating rapid hydrolysis of starch to glucose in salt tolerant M *Cicer arietinum* (Garg and Bharti, 2018). The activity of SPS as well as SS and AI increased simultaneously indicating that sucrose is synthesized upon salt stress, and concomitantly converted to glucose leading to increase in TSS concentration (Garg and Bharti, 2018).

The role of AM symbiosis in reinforcing osmotic adjustment in plants under salt stress via enhanced accumulation of osmolyte is now well-understood. However, these mechanisms are yet to be explicated at the molecular level. Therefore, directing future research in unraveling the molecular bases of osmolyte accumulation in M plants will help in all-inclusive understanding of the mechanisms.

### Oxidative Stress

Salinity mediated hyperosmotic and hyperionic stress induce another secondary stress in plants, called oxidative stress. It results due to the disturbance of equilibrium between ROS generation and its diminution by several antioxidants (Gill and Tuteja, 2010). ROS consists of a group of chemically reactive oxygen molecules such as hydroxyl radical (OH^-^), H_2_O_2_, .O_2_^-^, and O^2-^. ROS are generated as an after-effect of disengaged pathways in plant metabolism that causes the transfer of high energy electrons to molecular oxygen (Gill and Tuteja, 2010). Excessive generation of ROS disturbs various cell functions by attacking several biomolecules such as nucleic acid, protein, and membrane lipid (Foyer and Noctor, 2005). Salinity increases the level of lipid peroxidation (Evelin et al., 2012; Pedranzani et al., 2016; Zhang et al., 2018) resulting in higher membrane permeability and loss of ions from the cells (Estrada et al., 2013; Felicia et al., 2017).

**FIGURE 4:**
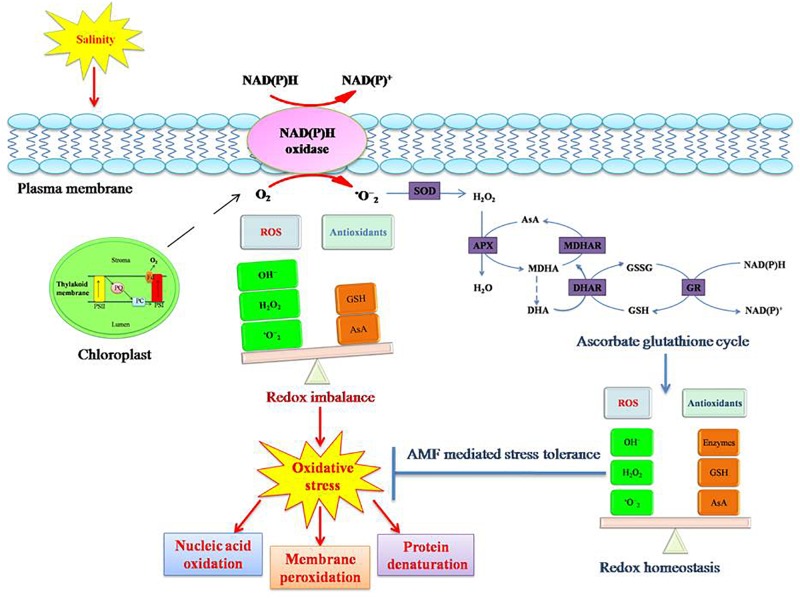
Salinity stress induced oxidative stress tolerance mechanism in plants. Salinity causes oxidative stress in plants due to redox imbalance resulting from disturbance in equilibrium between ROS (reactive oxygen species) and antioxidants. Increased ROS concentration in the cell results in protein denaturation, membrane peroxidation, and nucleic acid denaturation. This consequently disturbs the normal functioning of the cell. To counteract the adverse consequences, plants induce antioxidative pathways (enzymatic and non-enzymatic). Enzymatic antioxidants include superoxide dismutase (SOD), peroxidase (POX), catalase (CAT), ascorbate peroxidase (APX), monodehydroascorbate reductase (MDHAR), dehydroascorbate reductase (DHAR), and glutathione reductase (GR). Non-enzymatic antioxidants include ascorbate (AsA), glutathione (GSH), carotenoids, and α-tocopherol. An efficient antioxidative system abates the oxidative damage in plants. AM symbiosis reinforces the tolerance mechanism of plants to salinity induced oxidative stress.

Plants employ a two-pronged system to counteract the adverse consequences of ROS; enzymatic and non-enzymatic antioxidative systems. The enzymatic system consists of SOD, POX, CAT, APX, and GR. Non-enzymatic antioxidant molecules such as AsA, glutathione (GSH), carotenoids, and α-tocopherol also take part in quenching of toxic by-products of ROS (Gill and Tuteja, 2010). In fact, salinity tolerance in plants has been associated with the induction of antioxidative pathways and diminution of oxidative damage (Figure 4) (Porcel et al., 2012).

In the last few years, several studies have shown that one of the mechanisms AMF employ to alleviate salinity stress is an efficient ROS scavenging system. AMF augment the activities of antioxidant enzymes as well as increase the production of antioxidant molecules, ensuring a better ROS scavenging system. AMF colonization increases the concentrations of antioxidant molecules such as, α-tocopherols, AsA, GSH, and carotenoids in plants (Evelin and Kapoor, 2014). High α-tocopherol content limits auto-oxidation of lipids, thus preventing M plants from lipid peroxidation (Serbinova and Packer, 1994). High α-tocopherol content in M plants can be attributed to improved production of tocopheroxyl radicals, facilitated by the high AsA content (Thomas et al., 1992; Noctor and Foyer, 1998). Higher GSH levels and GR activity result in higher AsA levels in plants. GSH enables plants to directly scavenge more .O_2_^-^ and H_2_O_2_ as well as other ROS (Smirnoff, 1993; Briviba et al., 1998; Noctor and Foyer, 1998), and recycle more AsA via the AsA-glutathione cycle (Foyer and Halliwell, 1976). Carotenoids also prevent .O_2_^-^production (Viljanen et al., 2002; Ramel et al., 2012).

Mycorrhizal plants are reported to possess higher activities of enzymatic antioxidants under both saline and non-saline conditions. Elevated SOD activity was reported in M plants in comparison to their NM counterparts (Table 3). Higher SOD activity is associated with higher tolerance to salinity (Benavídes et al., 2000). Higher SOD enables detoxification of more O^2-^ to H_2_O_2_, that is further detoxified to H_2_O. The conversion of H_2_O_2_ to H_2_O can be catalyzed by enzymes, such as CAT, APX, and POX. Mycorrhizal plants are reported to have higher activities of H_2_O_2_ scavenging enzymes. Higher AsA concentration in M plants induces higher activity of APX, that is consecutively reliant on enhanced GR activity in M plants. This results in higher GSH pool, which ultimately depletes DHA concentration and recycles AsA (Foyer and Halliwell, 1976). Improved efficiency of GR maintains higher GSH:GSSG and NADP^+^:NADPH ratios and lowers superoxide radical production by sustaining the photosynthetic electron transport (Noctor and Foyer, 1998; Mittler, 2002). Higher activities of these enzymes in M plants are partially explained by better nutritional status. Mycorrhizal plants have been shown to have higher concentrations of micronutrients, such as Cu, Zn, Mn, and Fe. On the other hand, these enzymes are metalloenzymes whose activities are also governed by the availability of these micronutrients. However, the activities of these enzymes are dependent on various factors such as plant species, plant tissues, AMF species, level of salinity, and duration of stress.

**Table 3:** Some of the studies on effect of salinity and AMF on antioxidant response in plants.

S. No.	Salt level (mM NaCl)	Plant (Family)	Fungus^∗^	Parameters	Effects of		References
						
					Salinity	AMF on salt stressed plants	
1.	0, 50, 100	*Solanum lycopersicum* (Solanaceae)	*Glomus mosseae*	MDA content and CAT, SOD, POD, APX activities	Increased- MDA content, APX and POD activity. SOD and CAT activity increased at 50 mM and decreased at 100 mM.	Increased- enzyme activities Decreased- MDA content	Latef and Chaoxing, 2011
2.	0, 400	*Suaeda salsa* (Amaranthaceae)	*G. mosseae*	MDA content and activity of SOD and CAT	Increased- MDA content and activity of SOD and CAT	Further increased- SOD and CAT activity Decreased- MDA content	Li et al., 2012
3.	0, 50, 100, 200	*Trigonella foenum-graecum* (Fabaceae)	*Glomus intraradices*	Leaf and root MDA and H_2_O_2_ content, enzymatic and non-enzymatic antioxidants	Increased- leaf and root MDA, H_2_O_2_, AsA, α-tocopherol, GSH, carotenoid content; leaf and root SOD, APX, POD, GR activity and leaf CAT activity Decreased- root CAT activity at 200 mM	Increased- AsA, α-tocopherol, GSH, carotenoid content and SOD, APX, POD, GR, and CAT activity Decreased- leaf and root MDA and H_2_O_2_ content	Evelin and Kapoor, 2014
4.	0, 75, 150	*Sesbania sesban* (Fabaceae)	*Funneliformis mosseae, Rhizophagus intraradices*, and *Claroideoglomus etunicatum*	Enzymatic and non-enzymatic antioxidants.	Increased- SOD, CAT, APX, and GR activity; GSH and GSSG content Decreased- AsA content	Further increased the activity of enzymatic and non-enzymatic antioxidants	Abd-Allah et al., 2015
5.	0, 60, 80, 100	*Cicer arietinum* (Fabaceae)	*F. mosseae*	Ascorbate-glutathione cycle and antioxidant scavenging capacity	Increased- superoxide radical, H_2_O_2_, MDA, total ascorbate, AsA, DHA, total glutathione, GSH, and GSSG content; and SOD, CAT, GPOX, APX, MDHAR, DHAR, GR, and lipoxygenase activity,	Increased- CAT, GPOX, APX, SOD, MDHAR, DHAR, GR activity, further increase in GSG, GSSH, and total glutathione content Decreased- superoxide radical, H_2_O_2_, MDA content, and lipoxygenase activity	Garg and Bhandari, 2016a
6.	0, 200	*Digitaria eriantha* (Poaceae)	*Rhizophagus irregularis*	Root and shoot H_2_O_2_ and MDA content; antioxidant enzymes	Increased- root H_2_O_2_ content; root SOD, APX, GR activity; and shoot APX activity Decreased- root MDA content; shoot SOD, CAT, GR activity; and root CAT activity	Increased- shoot MDA content; shoot SOD, CAT, APX activity and root CAT and APX activity Decreased- root and shoot H_2_O_2_ content; root SOD; and root and shoot GR.	Pedranzani et al., 2016
7.	0, 60, 80, 100	*Cajanus cajan* (Fabaceae)	*F. mosseae* and *R. irregularis*	Root and shoot enzymatic and non-enzymatic antioxidants	Increased- GR, APX, SOD activity and MDA content Decreased- GSH:GSSG and AsA:DHA ratio	Increased- GSH:GSS and AsA:DHA ratio, further increased GR, APX, and SOD activity Decreased- MDA content	Pandey and Garg, 2017
8.	0, 200	*Cucumis sativus* (Cucurbitaceae)	*C. etunicatum, Rhizoglomus intraradices*, and *G. mosseae*	H_2_O_2_ and MDA content; enzymatic and non-enzymatic antioxidants.	Increased- total phenol, H_2_O_2_, and MDA content and activities of SOD, CAT, APX, and GR Decreased- AsA content	Increased- total phenol and AsA content and activities of SOD, CAT, APX, and GR Decreased- H_2_O_2_ and MDA content	Hashem et al., 2018


*^∗^Nomenclature of the fungus is as per the name used in the cited papers. MDA, malondialdehyde; CAT, catalase; SOD, superoxide dismutase; POD, peroxidase; APX, ascorbate peroxidase; GR, glutathione reductase; GSH, reduced glutathione; GSSG, oxidized glutathione; AsA, ascorbate; H_2_O_2_, hydrogen peroxide; DHA, dehydroascorbate; GPOX, glutathione peroxidase; MDHAR, monodehydroascorbate reductase; DHAR, dehydroascorbate reductase.*

Additionally, lower oxidative stress in M plants is also due to improved concentrations of osmolytes, such as proline, polyamine, and glycinebetaine (Pang et al., 2007; Evelin et al., 2012; Frosi et al., 2017). They bring about stabilization of sub-cellular components of cell membranes such as lipids and proteins, quenching of free radicals, and buffer cellular redox potential under salinity stress (Kishor et al., 1995; Yang et al., 2008).

### Water Status

High salt concentration in the rhizosphere also imposes physiological drought in plants. Salt immobilizes water and renders it unavailable for the plants (Füzy et al., 2008). Yang et al. (2014) observed that M *Malus hupehensis* seedlings maintained relatively higher leaf turgidity and lower leaf osmotic potential compared to NM plants, when subjected to salt stress. Chen et al. (2017) reported higher relative water content in M as compared to NM *Robinia pseudoacacia* plants under salt stress. This observation is explained by improved hydraulic conductivity of M plants attributed to AMF-induced altered root morphology, and the ability of M plants to explore macroelements well beyond the depletion zone facilitated by the extensive extramatrical mycelium of AMF (Schnepf et al., 2011). In addition, M plants also accumulate more compatible solutes to adjust the osmotic potential and enable efficient water usage by host plants (Graham and Syvertsen, 1984). Better water status in M plants may be explicated by the AMF regulated expression of aquaporin genes present in leaves and roots of salt stressed plants. However, each aquaporin gene in roots of M plants may respond differently to salt stress. For instance, in *Lycopersicon esculentum*, AMF colonization downregulated *LePIP1* gene (Ouziad et al., 2006) while the same gene was upregulated in *Lactuca sativa* (Jahromi et al., 2008). Recently, Chen et al. (2017) described the expression profile of aquaporin genes (*RpPIP1;1, RpPIP1;3, RpPIP2;1, RpTIP1;1, RpTIP1;3, RpTIP2;1*) in leaves and roots of M and NM *Robinia pseudoacacia* subjected to saline stress. Hence, the effect of AM symbiosis on transcription of aquaporin genes varies with the plant species, type of plant tissue they are expressed, and level of salinity. Furthermore, potential of different aquaporin genes to transport water and other solutes may differ and depend on their location in the cell (Ruiz-Lozano et al., 2012).

### Photosynthesis

Salt stress impedes photosynthesis and brings an enormous decline in crop productivity (Pitman and Läuchli, 2002; Sheng et al., 2008). Salt stress affects photosynthesis in several ways (Table 4). Salt-induced osmotic stress leads to lowering of leaf area, coupled with a decrease in stomatal and mesophyll conductance, which limits CO_2_ availability and assimilation (Chaves et al., 2009). This consequently decreases the supply of CO_2_ to RuBisCO. In addition to this, RuBisCO being sensitive to Cl^-^, loses its activity under salt stress (Seemann and Critchley, 1985). The decline in CO_2_ assimilation also leads to accumulation of excess energy that leads to increased accumulation of electrons in the thylakoid membranes. To dissipate this energy, PSII loses massive amount of electrons and causes injury of the photosynthetic tissues, which subsequently affects the net photosynthetic rate (Redondo-Gómez et al., 2010). Furthermore, salt stress has also been shown to degrade D1 and D2 proteins of PSII reaction center. These proteins are structural components of the PSII reaction center and play fundamental roles in phosphorylation of proteins coupled with flow of electrons (Jansen et al., 1996). A decline in the activity of photosynthetic pigment synthesizing enzymes is another way by which salt stress affects photosynthesis resulting in decreased concentration of photosynthetic pigments (Giri and Mukerji, 2004; Murkute et al., 2006; Sheng et al., 2008). The low concentration of chlorophyll may also be attributed to salt-induced low uptake of Mg^2+^ (Giri and Mukerji, 2004; Ibrahim et al., 2011), destruction of pigment-protein complexes due to salt-induced augmentation of chlorophyllase enzyme activity, and reduction of *de novo* protein synthesis (Levitt, 1980; Sultana et al., 1999; El-Tayeb, 2005).

**Table 4:** Some of the studies on effect of salinity and AMF on photosynthesis in plants.

S. No.	Salt level (mM NaCl)	Plant (Family)	Fungus^∗^	Parameters	Effects		References
						
					Salinity	AMF on salt stressed plants	
1.	0, 75	*Cucumis sativus* (Cucurbitaceae)	*Gigaspora rosea* BEG9	*F*v/*F*m and photosynthetic performance index	Decreased	Increased	Gamalero et al., 2010
2.	0, 100	*Citrus tangerine* (Rutaceae)	*Glomus mosseae* and *Paraglomus occultum*	Net photosynthetic rate, E, and G_s_	Decreased	Increased	Wu et al., 2010
3.	0, 50, 100, 200	*Capsicum annum* (Solanaceae)	*Glomus intraradices*	Chl content	Decreased	Increased	Beltrano et al., 2013
4.	0, 50, 100, 150, 200	*Medicago sativa* (Fabaceae)	*Glomus viscosum*	Chl content, G_s_, and stomatal density	Decreased	Increased	Campanelli et al., 2013
5.	0, 75, 150	*Oryza sativa* (Poaceae)	*Claroideoglomus etunicatum*	Plant gas-exchange parameters, photosynthetic pigments, RuBisCO activity and gene expression	Increased- iWUE, Φ_NPQ_, and expression of *rbcS* gene Decreased- G_s_, net photosynthetic rate, E, Φ_PSII_, and expression of *rbcL* gene	Increased- G_s_, net photosynthetic rate, E, iWUE, Φ_PSII_, RuBisCO activity Decreased- Φ_NPQ_ and expression of *rbcS* and *rbcL* gene	Porcel et al., 2015
6.	0, 75	*Populus cathayana* (Salicaceae)	*Rhizophagus irregularis*	Net photosynthetic rate, G_s_, C_i_, and E	Decreased	Increased- Net photosynthetic rate and C_i_ only	Wu et al., 2015
7.	0, 60, 80, 100	*Cicer arietinum* (Fabaceae)	*Funneliformis mosseae*	Photosynthetic pigments and RuBisCO activity	Decreased	Increased	Garg and Bhandari, 2016b
8.	0, 100, 200	*Leymus chinensis* (Poaceae)	*G. mosseae*	Plant gas-exchange parameters	Decreased- Net photosynthetic rate, C_i_, and G_s_	Increased- Net photosynthetic rate, C_i_, and G_s_	Lin et al., 2017
9.	0, 200	*C. sativus* (Cucurbitaceae)	*C. etunicatum, Rhizoglomus intraradices*, and *G. mosseae*	Photosynthetic pigments and G_s_	Decreased	Increased	Hashem et al., 2018
10.	0, 150, 300	*Zea mays* (Poaceae)	*Glomus tortuosum*	Plant gas-exchange parameters and photosynthetic pigments	Increased- Chl a:b, *F*_0_, and C_i_ Decreased- Chl a, Chl b, Chl a + b, *F*m, *F*v/*F*m, *F*v/*F*_0_, net photosynthetic rate, G_s_, E, and RuBisCO activity	Increased- Chl a, Chl b, Chl a + b, *F*m, *F*v/*F*m, *F*v/*F*_0_, net photosynthetic rate, G_s_, E, and RuBisCO activity Decreased- Chl a:b, *F*_0_, and C_i_	Xu et al., 2018


*^∗^Nomenclature of the fungus is as per the name used in the cited papers. *F*v/*F*m, quantum efficiency of photosystem II (PSII); E, transpiration rate; G_*s*_, stomatal conductance; Chl, chlorophyll; iWUE, intrinsic water use efficiency; RuBisCO, ribulose 1,6-bis phosphate carboxylase/oxidase; Φ_*NPQ*_, quantum yield of non-photochemical quenching; *rbcS*, gene encoding small RuBisCO subunit; *rbcL*, gene encoding large RuBisCO subunit; Φ_*PSII*_, quantum efficiency of photosystem II; C_*i*_, intercellular carbon dioxide content; *F*_*0*_, primary fluorescence; *F*m, maximal fluorescence; *F*v/*F*_*0*_, potential photochemical efficiency.*

Plants can safeguard the photosystems from light induced inhibition and damage in many ways. These include minimizing harvesting of light and dispersion of excess energy by non-photochemical quenching (NPQ) or cyclic electron flow (Lima Neto et al., 2015). AM symbiosis is known to bolster up these mechanisms and alleviate the negative effects of salinity on plant photosynthetic capacity (Talaat and Shawky, 2014; Porcel et al., 2015; Wu et al., 2015; Hidri et al., 2016; Chen et al., 2017). As compared to NM plants, M plants have higher net photosynthetic rate, more quantum yield (*F*v/*F*m; *F*v = *F*m − *F*_0_; *F*m = maximal fluorescence; *F*_0_ = minimal fluorescence), and actual quantum yield of PSII (Φ_PSII_) photochemistry under salt stress (Table 4) (Talaat and Shawky, 2014; Porcel et al., 2015; Wu et al., 2015; Hidri et al., 2016; Chen et al., 2017). AM symbiosis combats the negative effects of salt stress on photosynthesis in following ways – (i) improved water status in M plants results in maintaining larger leaf area and higher stomatal conductance, and consequently better assimilation of CO_2_ (Wu et al., 2015; Chen et al., 2017). Furthermore, aquaporins besides transporting water, can also promote diffusion of CO_2_ across plasma membrane of the mesophyll cells (Yang and Cui, 2009); (ii) ability of M plants to abate intercellular CO_2_ concentration (C_i_) ensures protection of the photosynthetic apparatus (Sheng et al., 2008; Chen et al., 2017). This is possible due to enhanced RuBisCO activity in M plants (Talaat and Shawky, 2014; Porcel et al., 2015; Garg and Bhandari, 2016b; Chen et al., 2017) owing to higher expression of the large subunit of RuBisCO, *RprbcL* gene (Chen et al., 2017). However, up regulation of gene expression may not always translate to increased RuBisCO activity due to delay in protein translation after gene transcription (Porcel et al., 2015); (iii) AM symbiosis empowers host plants to maintain the integrity of PSII by its prompt action to repair salt-induced degradation of D1 and D2 proteins. The genes encoding D1 (*RppsbA*) and D2 (*RppsbD*) were found to be up regulated by AM symbiosis under salinity conditions (Chen et al., 2017). This may also be due to higher concentration of polyamines and glycinebetaine in M plants (Pang et al., 2007; Talaat and Shawky, 2014). Glycinebetaine stabilizes PSII pigment-protein complexes as well as protect the activities of enzymes such as, RuBisCO and rubisco activase that are responsible for fixing CO_2_ (Talaat and Shawky, 2014). In fact, previous studies have reported that maintenance of PSII activity can help plants to adapt to abiotic stresses (Sheng et al., 2008; Hajiboland et al., 2010; Porcel et al., 2016; Hu et al., 2017); (iv) Mycorrhizal plants maintain higher chlorophyll and carotenoid concentration by improving the uptake of Mg^2+^ (Evelin et al., 2012; Hashem et al., 2015). Moreover, an increase in NPQ can limit *F*v/*F*m (Baker, 2008). However, M plants are reported to have lower NPQ (Baker, 2008). Thus, AM symbiosis enhances photosynthetic efficiency by proficient conversion of harvested light into chemical energy, and minimizing NPQ as compared to NM plants (Hu et al., 2017).

Furthermore, salinity also increases the activities of carbon metabolizing enzymes such as, pyruvate orthophosphate dikinase (PPDK), phospho-enol-pyruvate carboxylase (PEPC), NADP-MDH, and NAD-MDH (Hashem et al., 2015). Higher activities of these enzymes are required for better plant growth to overcome stress conditions (Doubnerová and Ryšlavá, 2011). Mycorrhizal plants were found to possess lower activities of these enzymes suggesting lower level of stress. On the other hand, salinity decreased the activity of NADP-ME and AMF colonization induced the activity of this enzyme (Hashem et al., 2015). Higher NADP-ME in M plants can increase carbon metabolism and contribute to stress tolerance (Hashem et al., 2015).

### Hormonal Regulation

Phytohormones regulate growth and development of plants under ambient as well as stressed conditions, and hence are also known as growth regulators (Fahad et al., 2015). They are derived from plant biosynthetic pathways and can act at the site of production or away from it. Phytohormones such as, ABA, auxin, BR, CK, GA, JA, SA, SLs, NO, and triazoles have been implicated to play significant roles in imparting salt-stress tolerance in plants (Fahad et al., 2015). In order to initiate suitable plant response to environmental stimulus, these hormones interplay amongst themselves to modulate biochemical and physiological processes that translate into mediation of growth, development, nutrient allocation, and source/sink transitions (Saeed et al., 2017). However, despite the large amount of literature available on phytohormones’ role in salinity tolerance, this aspect has found little interest in studies pertaining to AMF-mediated salinity tolerance in plants. Moreover, during AM symbiosis, ABA, auxin, JA, and SA are known to act as signaling molecules (Gutjahr and Paszkowski, 2009; Miransari, 2012). Therefore, it is postulated that these hormones play significant roles in improving plant tolerance to salinity stress.

Strigolactones consist of a new class of phytohormones that are involved in many aspects of plant development such as coordination of root growth and architecture according to the nutrient availability in soil, suppression of secondary branches in shoot, stimulation of internode length in a cross talk with auxin, regulation of leaf senescence, and induction of AM symbiosis (Agusti et al., 2011; Koltai, 2011; de Saint Germain et al., 2013; Yamada et al., 2014). They also play regulatory roles against abiotic stress. In order to exert its full effect, SLs need to modulate and interact with other phytohormones, especially auxin and ABA. In *Sesbania cannabina* seedlings, Kong et al. (2017) reported that H_2_O_2_ and SLs signaling are involved in AM mediated salt stress alleviation. Ren et al. (2018) observed that that in contrast to NM plants, AM colonization in *S*. *cannabina* maintained positive correlation between ABA and SLs. They proposed that AM colonization intensively altered ABA catabolism. This high ABA in M plants, protects it from salt stress by inducing the production of SLs via H_2_O_2_ signaling (Ren et al., 2018). On the perception of ABA signal, there is rapid production of H_2_O_2_ in the apoplast. The accumulation of H_2_O_2_ depends on NADPH oxidase activity, which also plays a significant role in ABA signaling (Kwak et al., 2003). This subsequently leads to increase in accumulation of SLs, and ultimately enhanced salt tolerance (Ren et al., 2018).

Exogenous applications of GA and SA have displayed boost in salinity tolerance in *Solanum lycopersicum* and *Cicer arietinum*, respectively (Khalloufi et al., 2017; Garg and Bharti, 2018). Foliar spray of GA_3_ resulted in better nutrient acquisition and manifold increase in GA concentration in M *Solanum lycopersicum* indicating that GA can promote salinity tolerance (Khalloufi et al., 2017). AMF colonization has also been shown to impart positive influence on the endogenous concentrations of GA (Shaul-Keinan et al., 2002). Seed priming with SA helped M *Cicer arietinum* to tolerate salinity stress by improving ionic homeostasis, modulating carbohydrate metabolism, and improving growth and yield. In fact, SA application also promoted AMF colonization (Garg and Bharti, 2018) and *vice versa* (Hashem et al., 2018). Salicylic acid, in conjunction with AMF have also been shown to improve salinity tolerance by reducing lipid peroxidation while increasing concentration of proline, proteins, reduced sugars, and K^+^ ion contents in shoots of *Ocimum basilicum* (Shekoofeh et al., 2012). In a recent study, Hashem et al. (2018) have reported an increase in concentration of JA in *Cucumis sativus* under salt stress. JA is a phytohormone belonging to octadecanoid family and is involved in plant’s response to biotic as well as abiotic stress. Pedranzani et al. (2016) also reported increased concentrations of JA, 12-OH-JA, and OPDA (precursor of JA) in *Digitaria eriantha* under salt stress. Their concentrations were further boosted in the presence of *Rhizophagus irregularis.* Thus, JA has also been implicated to play a key role in imparting salinity tolerance to plants. The underlying mechanisms of GA, SA, and JA in improving salinity tolerance in M plants are yet to be fully understood. Therefore, more studies are required to be directed toward AM-salinity experiments involving phytohormones.

## Challenges

This review highlights the mechanisms that AM symbiosis facilitates to impart salt stress tolerance in plants. However, there are several challenges that future research should address for comprehensive understanding of these mechanisms. The challenges are briefly discussed below:

(1) There is no debate on the role of AMF facilitating osmoregulation. However, studies pertaining to osmoregulation are mainly limited to the evaluation of concentrations of osmolytes. There is a need to elucidate these phenomena at the molecular level by investigating the genes that code for these molecules and their biosynthetic enzymes.
(2) AMF have been consistently shown to improve nutrient uptake and maintain ionic homeostasis. However, these reports are focused on determining the concentrations of nutrients in tissues and evaluating ratios, such as K^+^:Na^+^, Ca^2+^:Na^+^, and Ca^2+^:Mg^2+^. Significant progress has been made to understand the mechanisms involved in maintenance of K^+^:Na^+^ ratio by studying uptake and translocation of K^+^ and Na^+^ ions via transporters. However, there are very few reports that elucidate the uptake of nutrients at the molecular level. CNGCs are non-selective cation channels that take up K^+^, Na^+^, and Ca^2+^ (Kaplan et al., 2007). Till now, there are no investigations on CNGCs in M plants. Therefore, identification of ion transporters and expression studies can provide a better understanding of ion homeostasis in M plants.
(3) Sulfur is a major component of various molecules, such as cysteine, a sulfur donor in ABA synthesis; and glutathione, an antioxidant molecule that helps in detoxification of ROS, thereby helping plants cope with salinity stress. Plants absorb sulfur as sulfate via SULTR. It is also known that AMF can improve sulfur nutrition by enhanced uptake and its translocation to the root in low-sulfate environments (Allen and Shachar-Hill, 2009). However, no studies are available on the influence of AMF on plant sulfate uptake regulation or SULTR genes under saline conditions.
(4) Studies regarding the role of phytohormones are limited and inconclusive. For instance, far and few studies with respect to AMF-salinity have focused on ABA and SLs. Phytohormones, such as BR, JA, NO, and SA have been indicated to improve plant’s tolerance to salinity; however, they are yet to be explored as potential candidates that impart salinity tolerance to M plants. Moreover, SA and JA have been well documented as signals to alert neighboring plants upon biotic stress. On the other hand, M plants are well connected to each other owing to the existence of common mycorrhizal network. Therefore, future research should investigate if SA and JA play any role in eliciting stress signals to its neighbors.
(5) Under salt stress, lipid metabolism undergoes changes that can be associated with profound alterations in cell membrane integrity, composition, and function (Parihar et al., 2015). Though lipid peroxidation has been elucidated in AM-salinity studies, lipid metabolism in salt stressed M plants is yet to be investigated.
(6) Salt stress causes endocytosis (Hamaji et al., 2009). Earlier, Evelin et al. (2013) reported that NaCl treatment increased the incidence of endocytosis significantly in *Trigonella*. The vesicular surface increases the surface area for higher exchange capacity (K^+^ versus Na^+^), protecting plants from ionic toxicities, imbalances, or interactions between substances in the cytoplasm.
(7) Cell wall of a plant is the interface between plant and environment. Therefore, it is the first organelle that, senses, perceives, and responds to salt stress (Li et al., 2012; Van der Does et al., 2017). Hence, it is crucial that active cell wall integrity is preserved so as to allow plants to sense and respond to stress rapidly. Thus, it necessitates that the composition, biosynthesis of its components and organization into a membrane under stress conditions be well-informed. Therefore, future research should direct studies that evaluate how AMF influence the cell wall under saline conditions.

Directing future AM-salinity research to understand the above-mentioned challenges will immensely improve our understanding of the mechanisms of AMF facilitated salinity tolerance in host plants in order to obtain maximum benefit from AM symbiosis under salinity stress.

## Author Contributions

HE, TD, SG, and RK jointly conceptualized and wrote the manuscript, contributing major parts of the literature survey. All the authors have collectively reviewed the manuscript and approved it.

## Conflict of Interest Statement

The authors declare that the research was conducted in the absence of any commercial or financial relationships that could be construed as a potential conflict of interest.

## Abbreviations

ABA: abscisic acid
AI: acid invertase
AM: arbuscular mycorrhiza
AMF: arbuscular mycorrhizal fungi
APX: ascorbate peroxidase
AsA: ascorbate
BR: brassinosteroids
CAT: catalase
C_i_: intercellular CO_2_ content
CK: cytokinin
CNGCs: cyclic nucleotide-gated channels
Cu: copper
DHA: dehydroascorbate
*F*_0_: minimal fluorescence
FAO: Food and Agriculture Organization
*F*m: maximal fluorescence
*F*v/*F*m: quantum yield of photosystem II
GA: gibberellic acid
GR: glutathione reductase
GSH: reduced glutathione
GSSG: oxidized glutathione
H_2_O_2_: hydrogen peroxide
HATs: high affinity transporters
HKT: high affinity potassium transporter
JA: jasmonic acid
K: potassium
M: mycorrhizal
N: nitrogen
NaCl: sodium chloride
NAD-MDH: NAD-dependent malate dehydrogenase
NADP-MDH: NADP-dependent malate dehydrogenase
NADP-ME: NADP-malic enzyme
NHX: sodium/hydrogen exchanger
NM: non-mycorrhizal
NO: nitric oxide
NPQ: non-photochemical quenching
NR: nitrate reductase
.O_2_^-^: singlet oxygen
O^2-^: superoxide anions
OH: hydroxyl ions
P: phosphorus
*PC5S*: pyrroline-5-carboxylate synthase
PEPC: phospho-enol-pyruvate carboxylase
Pi: intrinsic phosphate concentration
PIP: plasma membrane intrinsic proteins
POX: peroxidase
PPBK: pyruvate orthophosphate dikanase
PSII: photosystem II
Put: putreseine
ROS: reactive oxygen species
SA: salicylic acid
SKOR: outward rectifying K^+^ channel
SLs: strigolactones
SOD: superoxide dismutase
SOS1: salt overly sensitive 1
Spd: spermidine
Spm: spermine
SPS: sucrose phosphate synthase
SS: sucrose synthase
SULTR: sulfate transporters
TIPs: tonoplast intrinsic proteins
TPP: trehalose-6-phosphate phosphatase
TPS: trehalose-6-phosphate synthase
TRE: trehalase
TSS: total soluble sugar
Zn: zinc
Φ_PSII_: quantum efficiency of photosystem II
